# Correlation between GRIK2 rs6922753, rs2227283 polymorphism and aggressive behaviors with Bipolar Mania in the Chinese Han population

**DOI:** 10.1002/brb3.1449

**Published:** 2019-10-20

**Authors:** Haibo Ma, Guanglei Xun, Renyun Zhang, Xiaohua Yang, Yu Cao

**Affiliations:** ^1^ Department of Psychiatry Shandong Mental Health Center Jinan China

**Keywords:** bipolar mania, GRIK2 gene, polymorphism, rs2227283, rs6922753

## Abstract

**Objectives:**

Animal studies have shown that glutamate receptor ionotropic kainate 2 (GRIK2) gene knockout mice are more impulsive and aggressive. This study aims to verify whether the rs6922753 and rs2227283 polymorphisms of the GRIK2 gene are associated with both aggressive behavior and bipolar mania in the Chinese Han population.

**Methods:**

Polymerase chain reaction (PCR) was applied in the genotype rs6922753 and rs2227283 polymorphisms of the GRIK2 gene in 201 bipolar manic patients with aggressive behaviors, 198 bipolar manic patients without aggressive behaviors, and 132 healthy controls. The Modified Overt Aggression Scale (MOAS) was used to evaluate aggressive behavior in patients with bipolar mania.

**Results:**

No correlation was found between aggressive behavior and the rs6922753 polymorphism in the three groups. The A/A genotype and A allele of the rs2227283 polymorphism were found significantly more frequently in patients with aggressive behavior than in healthy controls (*p* = .004 and *p* = .013, respectively) and in patients with nonaggressive behavior (*p* = .002 and *p* = .018, respectively). The A/A genotype and A allele were associated with an increased risk of aggressive behavior.

**Conclusion:**

This study suggests that the rs2227283 polymorphism of the GRIK2 gene is related to aggressive behaviors in bipolar manic patients and that the A/A genotype and A allele may increase the risk of the aggressive behavior in bipolar manic patients.

## Graphical abstract

This study suggested that the rs2227283 polymorphism of GRIK2 gene was related to the aggressive behaviors in bipolar manic patients, and A allele may increase the risk of the aggressive behavior in bipolar manic patients.

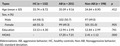

## 1 INTRODUCTION

Bipolar disorder (BD) is a chronic mental disorder characterized by the alternation of mania and depression, with a prevalence rate of approximately 0.8% worldwide (Ferrari et al., 2016). Aggressive behavior is a common symptom in psychiatry, and it can be classified into three categories among the patients who suffer from mental disorders in a broad sense: impulsive, segmental, and psychotic aggressive behavior (Hu, 2017). A large proportion of aggression in patients with bipolar disorder belongs to impulsive aggression since bipolar disorder patients have higher aggression scores than healthy controls (Ballester et al., 2012; Chou et al., 2013). Aggressive behavior is common among bipolar disorder, particularly during manic episodes (Belete et al., 2016). Aggression can be life‐threatening and causes serious social problems. Therefore, if the risk of aggressive behaviors with bipolar disorder is assessed and identified early, the risk of injury to patients and medical staff will be greatly reduced.

A general population survey found that a substantial proportion of aggressive behavior was caused by genetic factors (Barr & Driscoll, 2014; Takahashi & Miczek, 2014). Two large longitudinal samples of twin data showed that aggressive behavior was a stable trait with a heritability of 50% to 80% (Porsch et al., 2016), and other studies have found that there are more than 50 genes associated with aggressive behavior in mice (Maxson & Canastar, 2003; Miczek, Maxson, Fish, & Faccidomo, 2001). At present, research on genes related to aggression mainly involves serotonin system genes, dopamine system genes, monoamine oxidase genes, and brain‐derived neurotrophic factor genes. In addition, some studies have begun to focus on the glutamate receptor gene (Lutz, Marsicano, Maldonado, & Hillard, 2015), the nitric oxide synthase gene and other new candidate genes (Freudenberg, Carreno Gutierrez, Post, Reif, & Norton, 2016; Malki et al., 2016; Veroude et al., 2016). Although these new findings have led to a better understanding of the genetic mechanism of aggression, the molecular genetic mechanism of aggression is still unclear. Moreover, few studies have been conducted on the association between aggression and glutamate receptor genes in bipolar disorder patients.

Glutamate is an important excitatory neurotransmitter in the mammalian central nervous system; thus, it plays an important role in neuronal development and synaptic plasticity (Ribeiro, Vieira, Pires, Olmo, & Ferguson, 2017). Glutamate receptors, which belong to ligand‐gated ion channels, mediate rapid excitatory synaptic transmission through the central nervous system (Huettner, 2015). Glutamate receptors include metabotropic receptors and ionotropic receptors (Lagranha et al., 2014). The ionotropic glutamate receptor family can be divided into three groups: NMDA (N‐methyl‐D‐aspartate) receptors, AMPA (α‐amino‐3‐hydroxy‐5‐methyl‐4‐isoxazolepropionate) receptors, and KARs (kainate receptors) (Collingridge, Olsen, Peters, & Spedding, 2009). GRIK2, previously known as glutamate receptor 6 (GluR6), is a KARs. The GRIK2 gene is located in the 6q16.3 region, which is split into 17 exons and covers approximately 670 kb of the region (Barbon, Vallini, & Barlati, 2001). GRIK2 contributes to inhibitory transmission, regulates excitatory responses, and plays an important role in synaptic physiology (Barbon et al., 2001).

A recent study showed that the GRIK2 gene was likely to play a role in suicidal ideation in BD patients (de Sousa et al., 2017). Suicidal ideation is classified as auto aggression in the MOAS. Animal studies have shown that GRIK2 gene knockout mice were more impulsive and aggressive, suggesting that the GRIK2 gene has a unique role in controlling the behavioral symptoms of mania (Shaltiel et al., 2008). The rs6922753 and rs2227283 polymorphisms of the GRIK2 gene are located in the 5′ flanking region in exons 7 and 15, respectively. A previous genetic study provided further evidence that the rs6922753 and rs2227283 polymorphisms were associated with aggressive behavior in alcohol‐dependent patients of the Chinese Han population (Zou et al., 2014). Another study found that the rs6922753 and rs2227283 polymorphisms of the GRIK2 gene in schizophrenia were significantly related to aggressive behavior in the Chinese Han population (Wang et al., 2006). However, a recent study found that there was no association between aggression in bipolar disorder and the rs6922753 and rs2227283 polymorphisms of the GRIK2 gene in the Chinese Uyghur population (Zou et al., 2016). Thus far, only a few studies have focused on the relationship between the GRIK2 gene and aggressive behavior, and the results are not consistent.

The aim of this study was to examine the relationship between the GRIK2 rs6922753 and rs2227283 polymorphisms and aggressive behaviors with bipolar mania in the Chinese Han population. We tested the hypothesis that the rs6922753 and rs2227283 polymorphisms in patients with aggressive behavior differed from patients with nonaggressive behavior and healthy controls. We also assessed aggressive behavior of bipolar mania using the Modified Overt Aggression Scale (MOAS).

## 2 METHODS

### 2.1 Subjects

This study was conducted in strict accordance with the Declaration of Helsinki. All subjects of this study were Han nationality people and were recruited from outpatients and inpatients of the Shandong Mental Health Center, with the approval of its ethics committees. Meanwhile, all subjects of this study, including 399 bipolar manic patients and 132 healthy controls, have been completely informed and signed a consent form. The 399 patients included 201 patients with aggressive behavior and 198 patients with nonaggressive behavior. The patients met the criteria for bipolar mania according to the 10th revision of the International Classification of Diseases (ICD‐10), with at least two psychiatrists diagnosing bipolar mania at the same time. The clinical evaluation and data collection were performed by an experienced psychiatrist using structured clinical interviews and the MOAS. The patient inclusion criteria were as follows: (a) 18–60 years old, regardless of sex; (b) drug‐free or drug treatment stopped for at least 1 month. The patient exclusion criteria were as follows: (a) organic brain disease, physical disease or drug‐induced mental illness; (b) nervous system disease or other serious physical illnesses; (c) mental retardation; (d) drug abuse; and (e) pregnant or lactating women. The healthy controls included 132 healthy Han volunteers recruited in society and from hospitals who were matched with the patients by age, sex, and educational level. The control exclusion criteria were as follows: (a) psychiatric problems; (b) family history of mental illness; (c) nervous system disorder; and (d) acute or chronic physical illness.

### 2.2 MOAS assessment

The MOAS was introduced into China in 1991 (Xie & Zheng, 2001). There are four items in the scale, including verbal aggression, aggression against property, auto aggression, and physical aggression. The scale was divided into scores ranging from 0 to 4 according to the different severities of impulsive aggression, and the weighted score was calculated according to the verbal aggression score × 1, the aggression against property score × 2, the auto aggression score × 3, and the physical aggression score × 4. The total score is the sum of all weighted scores. The higher the score of each type of aggressive behavior, the stronger the aggression of this type of aggressive behavior. The MOAS showed satisfactory reliability and validity. The Cronbach's alpha values of each subscale ranged from 0.84 to 0.89 (Ho, 2006).

The MOAS scale was used to distinguish patients with aggressive behavior from those with nonaggressive behavior. Patients with aggressive behavior met the following criteria: a total MOAS score of ≥5 points and a physical aggression score of ≥1 point on the scale at the same time. Moreover, the patients with nonaggressive behavior met the following criteria: a total MOAS score of <5 points and a physical aggression score of 0 points.

### 2.3 DNA extraction and genotyping

For the rs6922753 polymorphism, the following primer sequences were used: 5′‐TTGCAAACCATCTACCACAAGTT‐3′ (forward primer) and 5′‐AGACAAGGCAGATGTTCTTCTCCT‐3′ (reverse primer). The sequence of the primers used for detection of the rs2227283 alleles was as follows: 5′‐CCTCCTCTCATCTTGCTAACC‐3′ (forward primer) and 5′‐CCCCCAGGGCACTGGCCTCTTTGCT‐3′ (reverse primer). Peripheral venous blood samples (5 ml) were collected in EDTA‐containing tubes. Genomic DNA was extracted from leukocytes with a blood genotyping kit (Tiangen Biotech). The extracted DNA samples were packed into three tubes and stored at −80°C for genotyping. Polymerase chain reaction (PCR) (Bah et al., 2004) was used to genotype the rs6922753 and rs2227283 polymorphisms of the GRIK2 gene. The total reaction volume of PCR was 50.0 μl, including 1 μl of DNA template, 1 μl of forward primer, and 1 μl of reverse primer. The polymerase activation began at 94°C for 5 min, followed by cycles including denaturation at 94°C for 30 s, annealing at 57°C for 30 s, and extension at 72°C for 45 s. A total of 35 cycles were performed, and after all the cycles were completed, the samples were extended at 72°C for 7 min. The case and control samples were genotyped in a random combination. In total, 5% of the samples were randomly selected for retesting, and the coincidence rate of retesting was 100%.

### 2.4 Statistical analysis

The data were statistically processed by SPSS 20.0 statistical software. Age, education level, and MOAS scores were analyzed as continuous variables. Continuous data were assessed for normality with the Kolmogorov–Smirnov test. These continuous variables were presented as the mean ± standard deviation. The goodness‐of‐fit *χ* ^2^ test was employed to calculate allele frequencies and genotypes and the frequency differences among patients with aggressive behavior, nonaggressive behavior and healthy controls. The goodness‐of‐fit *χ* ^2^ test was also used to assess Hardy–Weinberg equilibrium (HWE). A logistic regression was performed and analyzed to identify bipolar mania‐associated genotypes and allele frequencies by their odds ratios (ORs), 95% confidence intervals (CIs), and corresponding *p*‐values. Differences were considered significant when the *p*‐value <.05.

## 3 RESULTS

### 3.1 Demographics and scale characteristics

The three groups did not differ significantly in distributions of age, sex, and education levels. The total MOAS score in patients with aggressive behavior was significantly higher than that in patients with nonaggressive behavior (Table 1).

**Table 1:** Demographic information and scale characteristics

Types	HC (*n* = 132)	AB (*n* = 201)	Non‐AB (*n* = 198)	*p*
Age (mean ± *SD*)	33.74 ± 8.72	35.09 ± 9.16	34.84 ± 8.90	.412
Sex, *n* (%)				
Male	64 (48.5)	102 (50.7)	97 (49.0)	
Female	68 (51.5)	99 (49.3)	101 (51.0)	.905
Education	13.13 ± 4.30	12.94 ± 2.95	12.84 ± 2.99	.741
MOAS	–	17.20 ± 7.21	2.41 ± 1.12	.000

Abbreviations: AB, aggressive behavior; HC, healthy controls; Non‐AB, Nonaggressive behavior; *SD*, standard deviation.

### 3.2 Hardy–Weinberg equilibrium

The goodness‐of‐fit χ^2^ test showed that the rs6922753 and rs2227283 genotype distributions in patients with aggressive behavior (*χ* ^2^ = 1.633, *p* = .442; *χ* ^2^ = 1.378, *p* = .502), nonaggressive behavior (*χ* ^2^ = 0.053, *p* = .974; *χ* ^2^ = 2.847, *p* = .241), and healthy controls (*χ* ^2^ = 4.380, *p* = .112; *χ* ^2^ = 1.419, *p* = .492) were consistent with HWE.

### 3.3 Correlation of rs6922753 of the GRIK2 gene in the three groups

As shown in Table 2, the genotype and allele frequencies of rs6922753 in patients with aggressive behavior and nonaggressive behavior did not differ significantly from those in healthy controls. Moreover, the genotype and allele frequencies were not significantly different between patients with aggressive behavior and those with nonaggressive behavior (*p* > .05).

**Table 2:** Allele and genotype frequency distributions of rs6922753 of the GRIK2 gene in the three groups

	AB (*n* = 201) *n* (%)	HC (*n* = 132) *n* (%)	*p*	OR (95% CI)	Non‐AB (*n* = 198) *n* (%)	HC (*n* = 132) *n* (%)	*p*	OR (95% CI)	AB (*n* = 201) *n* (%)	Non‐AB (*n* = 198) *n* (%)	*p*	OR (95% CI)
Genotype												
T/T	76 (37.8)	56 (42.4)			67 (33.8)	56 (42.4)			76 (37.8)	67 (33.8)		
T/C	88 (43.8)	51 (38.6)	.334	0.79 (0.48–1.28)	95 (48.0)	51 (38.6)	.077	0.64 (0.39–1.05)	88 (43.8)	95 (48.0)	.365	1.23 (0.79–1.90)
C/C	37 (18.4)	25 (18.9)	.624	1.17 (0.63–2.15)	36 (18.2)	25 (18.9)	.410	1.29 (0.70–2.39)	37 (18.4)	36 (18.2)	.707	0.90 (0.52–1.55)
Allele												
T	240 (59.7)	163 (61.7)			229 (57.8)	163 (61.7)			240 (59.7)	229 (57.8)		
C	162 (40.3)	101 (38.3)	.598	0.92 (0.67–1.26)	167 (42.2)	101 (38.3)	.316	0.85 (0.62–1.17)	162 (40.3)	167 (42.2)	.591	1.08 (0.82–1.43)

Abbreviations: 95% CI, 95% Confidence Intervals; AB, aggressive behavior; HC, healthy controls; Non‐AB, Nonaggressive behavior; OR, Odds Ratios.

### 3.4 Correlation of rs2227283 of the GRIK2 gene in the three groups

Table 3 shows that the A/A genotype and A allele were found significantly more frequently in patients with aggressive behavior than in healthy controls (*p* = .004 and *p* = .013, respectively). The A/A genotype and A allele were also found to be significantly more frequent in patients with aggressive behavior than in patients with nonaggressive behavior (*p* = .002 and *p* = .018, respectively). The genotype and allele did not differ significantly between patients with nonaggressive behavior and healthy controls. The A/A genotype and A allele were associated with an increased risk of aggressive behavior (OR = 2.03, 95% CI = 1.25–3.30; OR = 1.50, 95% CI = 1.01–2.07; OR = 1.96, 95% CI = 1.28–3.01; OR = 1.42, 95% CI = 1.06–1.89, respectively).

**Table 3:** Allele and genotype frequency distributions of rs2227283 of the GRIK2 gene in the three groups

	AB (*n* = 201) *n* (%)	HC (*n* = 132) *n* (%)	*p*	OR (95% CI)	Non‐AB (*n* = 198) *n* (%)	HC (*n* = 132) *n* (%)	*p*	OR (95% CI)	AB (*n* = 201) *n* (%)	Non‐AB (*n* = 198) *n* (%)	*p*	OR (95% CI)
Genotype												
A/A	95 (47.3)	41 (31.1)			64 (32.3)	41 (31.1)			95 (47.3)	64 (32.3)		
A/G	81 (40.3)	71 (53.8)	.004	2.03 (1.25–3.30)	107 (54.0)	71 (53.8)	.889	1.04 (0.63–1.70)	81 (40.3)	107 (54.0)	.002	1.96 (1.28–3.01)
G/G	25 (12.4)	20 (15.2)	.789	0.91 (0.47–1.78)	27 (13.6)	20 (15.2)	.741	1.12 (0.58–2.14)	25 (12.4)	27 (13.6)	.521	0.82 (0.44–1.51)
Allele												
A	271 (67.4)	153 (58.0)			235 (59.3)	153 (58.0)			271 (67.4)	235 (59.3)		
G	131 (32.6)	111 (42.0)	.013	1.50 (1.01–2.07)	161 (40.7)	111 (42.0)	.722	1.06 (0.77–1.45)	131 (32.6)	161 (40.7)	.018	1.42 (1.06–1.89)

Abbreviations: 95% CI, 95% Confidence Intervals; AB, aggressive behavior; HC, healthy controls; Non‐AB, Nonaggressive behavior; OR, Odds Ratios.

## 4 DISCUSSION

In this study, we examined the relationship between the GRIK2 gene and aggressive behavior in patients with bipolar disorder. No correlation was found between the genotype and allele of rs6922753 and aggressive behavior in the three groups. Similarly, previous studies also failed to discover differences in the rs6922753 polymorphism between bipolar disorder patients with aggressive behavior and patients with nonaggressive behavior in the Chinese Han population (Hu, Zou, Zhao, Tong, & Zhang, 2017). Inconsistent with the above studies, one study reported that the rs6922753 polymorphism was associated with aggressive behavior in a study on domestic violence in patients with alcohol dependence in a Chinese Uyghur population (Zhang, Tong, Zou, Zhao, & Dong, 2016). Meanwhile, previous studies revealed that GRIK2 gene knockout mice were more active in several experiments and also had more aggressive displays compared with wild‐type mice (Shaltiel et al., 2008). The contradiction between this study and the previous studies likely results from the racial differences. On the one hand, bipolar disorder is a heterogeneous disease with different classifications and clinical manifestations. On the other hand, the results may be affected by differences in the criteria for sample selection or the accuracy of evaluating patient behavior.

In the present study, the A/A genotype and A allele of the rs2227283 polymorphism were identified significantly more frequently in patients with aggressive behavior than in healthy controls and patients with nonaggressive behavior. Thus, the A/A genotype and A allele were associated with an increased risk of aggressive behavior. This report is known to be the first research on the correlation between the rs2227283 polymorphism and aggressive behaviors in patients with bipolar mania. Another study reported that the rs2227283 polymorphism of the GRIK2 gene in schizophrenia was significantly related to aggressive behavior, and the A allele was a risk factor for aggressive behavior in the Chinese Han population (Wang et al., 2006). In addition, another study showed that the rs2227283 polymorphism of the GRIK2 gene had relevance to aggressive behavior in alcohol‐dependent patients of the Han population, and the A allele was a risk factor for aggressive behavior (Zou et al., 2014). However, a previous study reported that no correlation was found between aggressive behavior and the rs2227283 polymorphism in patients with bipolar disorder in the Uyghur population (Zou et al., 2016).

The mechanism of how the rs2227283 polymorphism affects aggressive behavior remains unclear. A recent study suggested that glutamate plays a catalytic role in aggressive behavior. The hypothesis is that glutamate may enhance defense‐anger behavior by stimulating NMDA, AMPA, and metabotropic receptors in areas such as the amygdala and hypothalamus (Comai, Tau, Pavlovic, & Gobbi, 2012). However, a previous study revealed that the levels of glutamate receptor ionotropic kainate 1 (GRIK1) and kainate 2 (KA‐2) receptors in the hippocampus and prefrontal cortex were decreased in GRIK2 gene knockout mice (Shaltiel et al., 2008). The kainate receptors (KARs) are composed of five subunits: GRIK1‐3 and KA‐1‐2 receptors (also named GRIK1‐5) (Traynelis et al., 2010). KARs are expressed in neuronal circuits related to emotion regulation (Shi et al., 2018). KARs are involved in the regulation of excitatory postsynaptic currents and modulate transmitter release at both the excitatory and inhibitory synapses (Møllerud, Frydenvang, Pickering, & Kastrup, 2017; Wilding & Huettner, 2019). These aggression behaviors are likely caused by the obstruction of the transport pathway of GRIK1 and the KA‐2 receptors. Some studies have found that a small group of molecules associated with the GRIK2 gene play an important role in the mechanism of controlling manic symptoms (Beneyto, Kristiansen, Oni‐Orisan, McCullumsmith, & Meador‐ Woodruff, 2007; Kato, Kubota, & Kasahara, 2007). These molecules include ERK1 (Frey et al., 2013; Sodersten et al., 2014), GSK‐3b (Kim, Won, & Yoon, 2019), mtDNA polymerase (Schulmann et al., 2019), and CLOCK (Etain, Milhiet, Bellivier, & Leboyer, 2011). Whether these molecules act independently or together to control behaviors associated with manic symptoms requires further confirmation. These results suggest that the GRIK2 gene plays a novel role in controlling aspects related to manic behavior symptoms, such as aggression, irritability and psychomotor excitement. This study showed that the rs2227283 polymorphism of the GRIK2 gene in people from the Han population may be associated with the occurrence of aggression in bipolar disorder, and the A allele was a risk factor in aggressive behavior. Moreover, the glutamate system may be involved in the pathophysiological mechanism of aggressive behavior.

Some limitations of the present study should be considered. First, the sample size in this study was relatively small. Although the results of this study found that the rs2227283 polymorphism was associated with aggressive behavior in bipolar manic patients, false‐positive results should not be ruled out. Second, some mutations in the GRIK2 gene may be related to the symptoms, diagnostic subtypes and family history of bipolar disorder. Due to the lack of relevant information, this study is unable to assess the impact of these factors on the interactions between the rs6922753 and rs2227283 polymorphisms and bipolar disorder. Finally, the biological mechanism of rs2227283 associated with aggressive behavior in bipolar manic patients remains unclear, although this study found that the gene locus is associated with aggressive behavior in patients with bipolar mania. Therefore, further studies are necessary to better determine the biological mechanism of the rs2227283 polymorphism associated with aggressive behavior of bipolar disorder.

## 5 CONCLUSION

In summary, the data of this study support the association between the rs2227283 polymorphism and aggressive behavior in bipolar manic patients, and the current research provides useful information for further study on the relationship between the GRIK2 gene and aggressive behavior. This is the initial report to support a significant association between the rs2227283 polymorphism and aggressive behavior in bipolar mania in the Han nationality in China. Future studies should extend these findings to a larger Chinese Han population and other racial and ethnic groups to confirm their associations. Meanwhile, the precise biological mechanism of rs2227283 associated with aggressive behavior in bipolar manic patients should also be identified in the future.

## CONFLICT OF INTEREST

All authors have stated that they have no conflicts of interest.

## AUTHOR CONTRIBUTION

Yu Cao designed this study. Renyun Zhang and Xiaohua Yang performed the data collection and performed data analyses. Haibo Ma and Guanglei Xun were responsible for manuscript writing. All authors contributed to discussing the results. All authors read and approved the final manuscript.

## Data Availability

The data that support the findings of this study are available on request from the corresponding author. The data are not publicly available due to privacy or ethical restrictions.
